# Lifespan Changes in Functional Brain Networks: A Study Using fMRI from Three Human Connectome Project Studies and Alzheimers Disease Neuroimaging Initiative

**DOI:** 10.1002/alz70862_110101

**Published:** 2025-12-23

**Authors:** Yaotian Wang, Shuoran Li, Jie He, Lingyi Peng, Qiaochu Wang, Xu Zou, Dana L Tudorascu, David J Schaeffer, Lauren K Hayrynen Schaeffer, Diego Szczupak, Jung Eun Park, Stacey J Sukoff Rizzo, Gregory W Carter, Afonso C Silva, Tingting Zhang

**Affiliations:** ^1^ Emory University, Atlanta, GA USA; ^2^ University of Pittsburgh, Pittsburgh, PA 15213‐2921, PA USA; ^3^ University of Pittsburgh, Pittsburgh, PA USA; ^4^ Department of Psychiatry, University of Pittsburgh, Pittsburgh, PA USA; ^5^ University of Pittsburgh School of Medicine, Pittsburgh, PA USA; ^6^ The Jackson Laboratory, Bar Harbor, ME USA

## Abstract

**Background:**

Understanding lifespan changes in functional connectivity (FC) within the human brain is critical for advancing both scientific knowledge and clinical applications, particularly for identifying abnormal patterns associated with Alzheimer’s disease (AD). Although numerous studies have indicated significant changes in FC and cognitive functions across the lifespan, inconsistent results have surfaced. This is largely due to the small sample sizes used in previous studies and the intricate nature of age‐related changes in brain functional networks.

**Method:**

To address these challenges, this study utilized functional magnetic resonance imaging (fMRI) data aggregated from three Human Connectome Project (HCP) studies: HCP‐Young Adults, HCP‐Development, and HCP‐Aging. We developed a novel clustering‐enabled regression method to analyze the aggregated data. This method identified clusters of brain regions exhibiting similar age‐related FC trajectories and uncovered diverse patterns of changes across different region clusters. Furthermore, we evaluated FC differences among healthy individuals, subjects with mild cognitive impairment (MCI), and those with AD.

**Result:**

While most brain connections between pairs of regions show minimal yet statistically significant FC changes with age, only a tiny proportion of connections exhibit practically significant age‐related changes in FC. Among these connections, FC between region clusters from the same functional network tends to decrease over time, whereas FC between region clusters from different networks demonstrates various patterns of age‐related changes. Moreover, our research uncovers sex‐specific trends in FC changes. Females show much higher FC mainly within the default mode network, whereas males display higher FC across several more brain networks.

Our analysis also revealed significant differences in how FC deviates from the norm in subjects with MCI and AD. Females with MCI or AD, on average, show more connections with deviated FC compared to males. Additionally, AD subjects exhibit more altered connections than those with MCI.

**Conclusion:**

Leveraging an extensive dataset from three combined HCP studies, our research offers fresh perspectives on the heterogeneity and complexity of age‐related FC changes, challenging prior studies that relied on smaller datasets. The detailed understanding of these patterns is critical for advancing our grasp of brain function changes throughout the lifespan and improving clinical assessments of neurodegenerative conditions.

